# Sociodemographic and clinical characteristics of children with tic disorders and behavioral problems: A real-world study and development of a prediction model

**DOI:** 10.1186/s12887-023-03864-y

**Published:** 2023-02-02

**Authors:** Fang Liu, Gaohua Wang, Jingping Ye, Baozhen Yao, Junling Wang, Huaqian Wang, Hong Liu

**Affiliations:** 1 Department of Pediatrics, Renmin Hospital of Wuhan University, Wuhan, 430060 China; 2 Department of Psychiatry, Renmin Hospital of Wuhan University, Wuhan, 430060 China

**Keywords:** Behavioral problems, Sociodemographic characteristics, Clinical characteristics, Tic disorders, Children, Nomogram

## Abstract

**Background:**

Tic disorders (TD) are complex neuropsychiatric disorders frequently associated with a variety of comorbid problems, whose negative effects may exceed those of the tics themselves. In this study, we aimed to explore the sociodemographic and clinical characteristics of children with TD and behavioral problems, and develop a prediction model of behavioral problems based on the predictors under real-world conditions.

**Methods:**

A hospital-based cross-sectional study was conducted on children with TD. Behavioral problems were surveyed using the Achenbach Child Behavior Checklist (CBCL). Sociodemographic information was collected from face-to-face interviews using an electronic questionnaire administered during the initial ambulatory visit. Clinical data were collected from medical records, and quality control was performed. The sociodemographic and clinical characteristics of patients with and without behavioral problems were statistically compared, and a nomogram prediction model was developed based on multivariate logistic regression analysis. The discriminatory ability and clinical utility of the nomogram were assessed by concordance index (C-index), receiver operating characteristic (ROC) curve, decision curve analysis (DCA) and clinical impact curve (CIC).

**Results:**

A total of 343 TD cases were included in the final analysis, of which 30.32% had behavioral problems. The prediction model showed age 12–16 years, abnormal birth history, parenting pattern of indulgence, parent/close relatives with psychiatric disorders, chronic motor or vocal tic disorder (CTD)/Tourette syndrome (TS) and moderate/severe tic severity were associated with behavioral problems in children with TD. The C-index of the prediction model (nomogram) was 0.763 (95% confidence interval, 0.710 ~ 0.816). The nomogram was feasible for making beneficial clinical decisions, according to the satisfactory results of the DCA and CIC.

**Conclusions:**

A nomogram prediction model for comorbid behavioral problems in children with TD was established. The prediction model demonstrated a good discriminative ability and predictive performance for beneficial clinical decisions. This model further provides a comprehensive understanding of associated sociodemographic and clinical characteristics by visual graphs and allows clinicians to rapidly identify patients with a higher risk of behavioral problems and tailor necessary interventions to improve clinical outcomes.

## Introduction

Tic disorders (TD) are complex neuropsychiatric disorders with onset before the age of 18 years, characterized by the presence of repetitive, involuntary, nonrhythmic, sudden movements, or vocalizations that can involve discrete muscle groups [1, 2]. According to the DSM-5 [3], TD encompass provisional tic disorder (PTD), chronic motor or vocal tic disorder (CTD), and Tourette syndrome (TS). PTD is considered when individuals have at least one motor tic and/or vocal tic, with a disease duration of less than one year. CTD is diagnosed when individuals have single or multiple motor or vocal tics, but not both appear at the same time during the course of the disease, with a duration of longer than one year. TS is diagnosed when individuals have multiple motor tics and one or more vocal tics, which may not appear at the same time, with a disease duration of more than one year. Tics tend to follow an unpredictable waxing and waning pattern over time, and could persist into adulthood [4]. Although the typical natural history is of improvement or remission over time, TD are frequently associated with a variety of comorbid problems whose negative effects may exceed those of the tics themselves [2, 5]. About 85–88% of individuals with TS have been reported at least one comorbid disorder, with the most common being attention-deficit/hyperactivity disorder (ADHD) and/or obsessive–compulsive disorder (OCD) [6–8]. Other disorders such as anxiety/depression disorders, learning disorder, oppositional defiant disorder, disruptive behavior disorders, externalizing disorders and autism spectrum disorders are also observed [9, 10]. Among these, the presence of comorbid behavioral problems is very common and can cause significant adverse effects on quality of life, and should therefore be considered in such patients [11, 12].

Behavioral problems include, but are not restricted to, withdrawal, hyperactivity, aggression, disruptive behavior, depression, and schizoid [11]. TD and comorbidities typically present deficits in inhibition, characterized by inattention, hyperactivity, aggression, obsessive–compulsive and other behavioral problems [13, 14]. Biological, psychological, and socio-environmental factors can contribute to the occurrence and persistence of TD and comorbidities. Previous studies have confirmed several factors, including genetic factors, parental psychiatric disorders, prenatal and perinatal epigenetic factors, family structure, poor parental relationships, and abnormal immune responses, are linked to TD development [9, 15–17]. However, most of those published studies were limited to investigating one factor or one category of factors, and there is lack of a predictive model specifically designed for behavioral problems in TD patients and the ability to draw conclusions about the most important predictors in an inclusive model and the combined impact of these predictors. Furthermore, to date, no published studies have yet developed practical predictive tools to examine the impact of sociodemographic and clinical characteristics. Knowledge of the impact of sociodemographic and clinical characteristics on behavioral problems would be helpful to clinicians in tailoring treatment interventions, and ultimately improve the quality of care for children with TD. Nomograms have been used to provide individualized evaluation of the clinical event incidence on many occasions and as a reliable statistical tool to create a simple intuitive graph to quantify the risk of a clinical event [18–20]. It is typically constructed based on multivariate regression analysis and transforms complex regression equations into visual graphs, to exhibit the combined impact of variables in the prediction model. To develop a model for predicting the risk of behavioral problems in TD patients and to provide a quantitative predictive tool for early clinical screening of individualized risk of TD with behavioral problems have high clinical application value.

In this study, we used a hospital-based database to explore the sociodemographic and clinical characteristics of children with TD and behavioral problems under real-world conditions, relative to children with TD without behavioral problems. We aimed to develop a nomogram prediction model based on independent predictors to examine the impact of sociodemographic and clinical characteristics on behavioral problems in children with TD.

## Material and methods

### Study population

This was a hospital-based cross-sectional study. We consecutively enrolled children aged 4–16 years who first visited the Department of Pediatrics, Renmin Hospital of Wuhan University from July 2019 to December 2021 with a diagnosis of TD. This hospital is one of the largest public tertiary hospitals in the capital city of Hubei province, with vast clinical services and a technological ability ranking among the top in China. Children were excluded for the following reasons: (1) had confirmed the following adverse mental or nervous conditions before the initial visit: schizophrenia, bipolar disorder, autism spectrum disorders, ADHD, OCD, intellectual disability and epilepsy; and (2) incomplete clinical data, incorrect information or loss of contact.

### Data collection

#### Sociodemographic characteristics: personal, parental and family-environmental factors

Sociodemographic characteristics include individual characteristics and socio-environmental influences. During the initial ambulatory visit of the patient and his/her main caregiver, sociodemographic information was collected through face-to-face interviews using an electronic questionnaire. This allowed us to generate variables that measured the child’s sociodemographic characteristics, including (1) personal factors, including gender, age, gestational age (GA), birth weight (BW), mode of delivery, birth history, and underlying diseases; (2) parental factors, including maternal age at pregnancy, method of feeding (within 6 months after birth), paternal and maternal education level; and (3) family-environmental factors, including family structure, parenting pattern, parental involvement in care, family relationship, family history of TD or psychiatric disorders.

The selection and evaluation of factors mainly refers to general appellations in sociology and previous literature [4, 9]. Nuclear family refers to those which include parents and children only, while stem family refers to those which include parents, unmarried children, married sons and their wives. Parenting patterns were assessed via face-to-face interviews with questions about different parent behaviors occurred in the last four weeks (e.g.,“You have rules that your child must follow”,“You listen to what your child to say”,“You punish your child when he/she fails to meet your demand”). Based on the caregiver’s report, four parenting patterns were categorized as: democratic (appropriate on both demand and responsiveness), slant scold (high demand and punish often), slant interference (high demand and low responsiveness), and indulgence (low demand or low on both demand and responsiveness). Family relationship was divided into harmony and inharmony, according to the self-evaluation of the caregivers. Abnormal birth history included, but was not restricted to, asphyxia, hypoxia, neonatal pneumonia, intracranial hemorrhage, and hyperbilirubinemia during prenatal and perinatal periods. Underlying diseases included, but were not restricted to, allergic rhinitis, sinusitis, asthma, severe eczema, recurrent respiratory infections, conjunctivitis, enuresis, and encephalitis. Physicians who conducted the interviews were trained uniformly. Senior researchers checked the collected data to perform the quality control.

#### Clinical characteristics: TD type, tic form and tic severity

The diagnosis of tics was based on the patient’s clinical history, video recording of tics, and complete physical examination with direct clinical observation. The clinical characteristics of tics include: (1) TD type: PTD, CTD and TS; (2) tic form: simple (motor/vocal tics) and complex, depending on the duration of tics and part(s) of the body and group(s) of muscles involved; (3) tic severity: mild, moderate and severe, usually evaluated with the Yale Global Tic Severity Scale (YGTSS), a clinician-rated semi-structured interview with demonstrated reliability and validity (Cronbach's α coefficient reaching 0.9) [21]. It elicits the characters of number, frequency, intensity, complexity, and interference of motor and vocal symptoms with 0–5 score (evaluated motor and vocal symptoms separately, maximum rating 50), and also produces a total impairment score (range: 0–50). The total YGTSS score (maximum rating 100) is obtained by summing up the scores. The criteria for determining severity are as follows: Total YGTSS score < 25, mild; 25–50, moderate; and > 50, severe [4]. Clinical diagnosis information was extracted from the medical records. Due to the small theoretical frequency of cases, some adjacent groups with similar properties were combined in our study (CTD and TS, simple motor tics and simple vocal tics, and moderate and severe).

#### Behavioral problems evaluated by Achenbach Child Behavior Checklist (CBCL)

CBCL is a widely used, validated, parent-rated scale assessing the frequency and intensity of behavioral and social competence of children and adolescents [22]. The CBCL-Chinese (version of the CBCL translated into Chinese and standardized in China) contains various subscales for different ages and genders [23]. CBCL 4–16 is composed of 113 items on a 3-point scale (0 = not true; 1 = somewhat or sometimes true; 2 = very true or often true) to allow computation of scores. This scale measures problematic behaviors over the previous six months, which grouped into internalizing problems (anxiety, depression, withdrawal, and somatic complaints), externalizing problems (aggressive behaviors, hyperactivity, and delinquent behaviors), and total behavioral problems. The score of any subscale or the total scale score equal to or above the 98th percentile was considered abnormal, indicating behavioral problems [23]. In our study, all caregivers completed the CBCL scale during the first clinical evaluation. Most of them completed without difficulty, and assistance including clear explanations was provided if needed.

### Statistical analyses

Statistical analyses were performed with R software version 3.5.2 (R Foundation for Statistical Computing, Vienna, Austria. http://www.r-project.org/). All categorical data are shown as frequencies and proportions, and were compared by Chi-square test (*χ*^*2*^ test) or Fisher’s exact test. Selected sociodemographic and clinical characteristics were compared between TD patients with and without behavioral problems. Univariate (unadjusted)and multivariate (adjusted) logistic regression analyses were performed to estimate the effects of the factors mentioned above on the behavioral problems of children with TD. The selected factors were examined by multiple collinearity diagnostics before multivariate logistic regression analyses (diagnostic criteria: the variance inflation factor value > 5 and tolerance < 0.1) [24, 25]. Association estimates were indexed as odds ratios (ORs) and 95% confidence intervals (95%CI) for the explanatory variables. *P* < 0.05 was considered statistically significant (two-tailed). The prediction model of TD with behavioral problems based on the logistic regression was plotted as a nomogram, and its discriminatory ability was evaluated by concordance index (C-index) and area under curve (AUC) from a receiver operating characteristic (ROC) curve analysis. The clinical utility of the nomogram was assessed by decision curve analysis (DCA) and clinical impact curve (CIC) [26].

## Results

### Sociodemographic and clinical characteristics of TD with or without behavioral problems

**Fig. 1:**
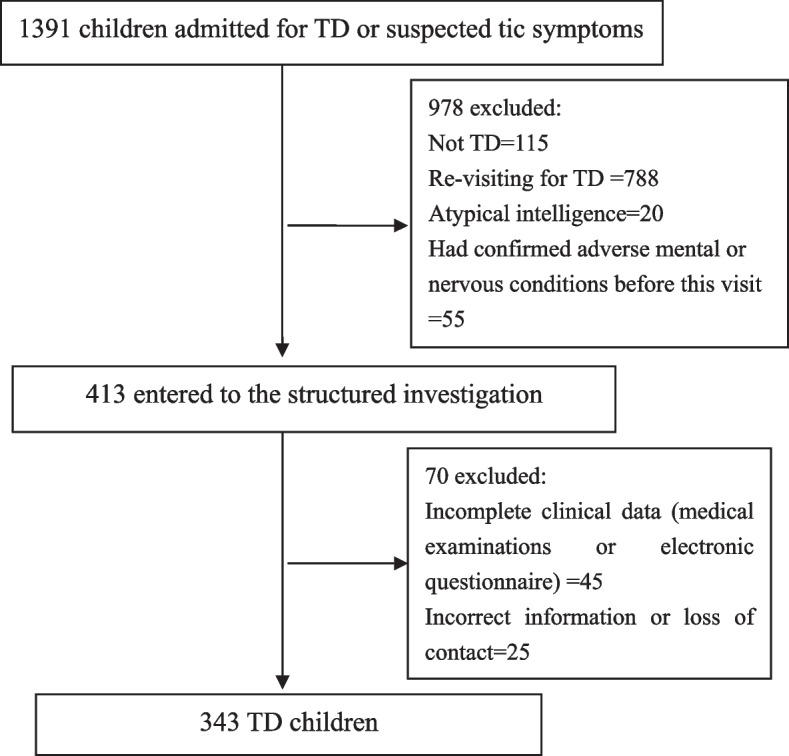
Flow diagram of the study sample

**Fig. 2:**
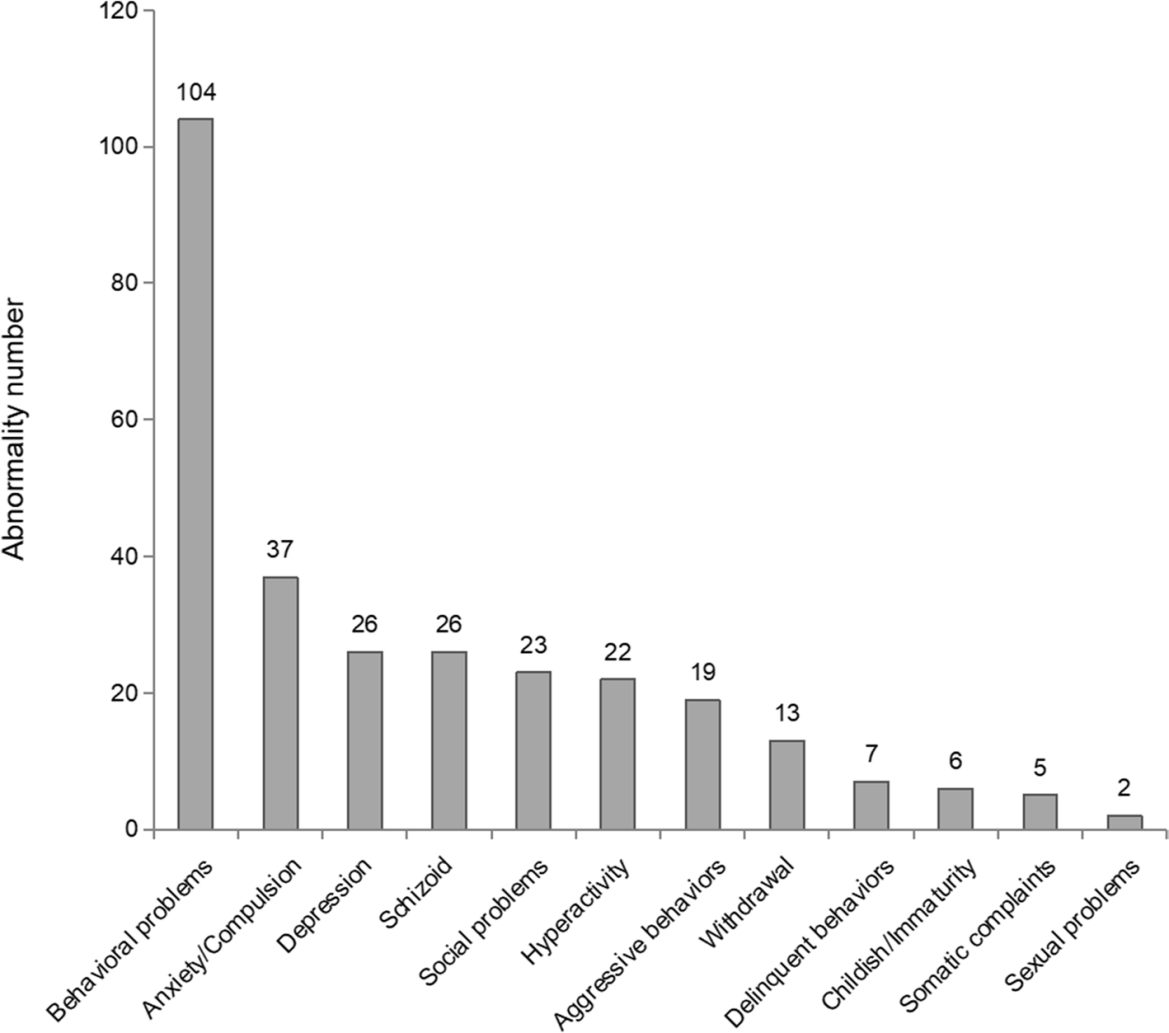
Numbers of TD patients with abnormal scores of CBCL main subscales and behavioral problems

**Fig. 3:**
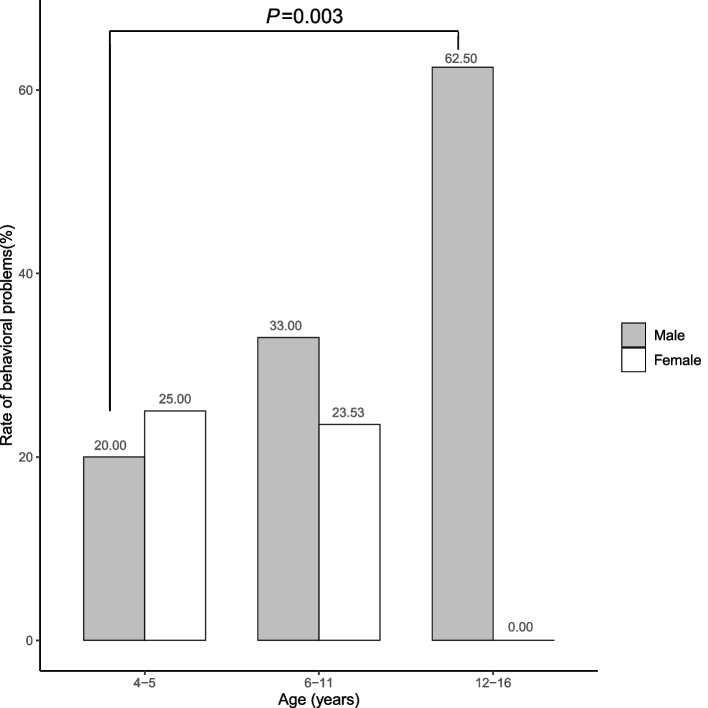
The rates of behavioral problems among different genders and ages in children with TD

Among the 1391 children with the chief complaint of TD or suspected tic symptoms, 343 sequential cases of TD (7.88 ± 2.26 years) were identified for the final analysis after screening the medical records and electronic questionnaires (Fig. 1). According to the CBCL scale, 104 (30.32%) patients had behavioral problems (Fig. 2). Male patients had a higher rate of behavioral problems than female patients, although this difference was not statistically significant (male: 88/274, 32.12%; female: 16/69, 23.19%; *P* = 0.195). The rate gradually increased with age in male patients, whereas the opposite trend was observed in female patients. The difference between male children aged 4–5 years and aged 12–16 years was significant (Fig. 3).

The sociodemographic and clinical characteristics of the children with and without behavioral problems are presented in Table 1. Those exhibiting behavioral problems were more likely to be aged 12–16 years, have an abnormal birth history, and have a parenting pattern of slant scold/indulgence, inharmony family relationship, or parent/close relatives with psychiatric disorders. Children with CTD/TS, complex tics or moderate/severe tic severity also tend to have behavioral problems.

**Table 1:** Sociodemographic and clinical characteristics of study subjects with and without behavioral problems

	Total (*N* = 343)	TD with behavioral problems (*N* = 104)	TD without behavioral problems (*N* = 239)	*P*
**Personal factors, n (%)**				
Gender				0.195
Male	274(79.88)	88(84.62)	186(77.82)	
Female	69(20.12)	16(15.38)	53(22.18)	
Age (years)				0.015
4–5	71(20.70)	15(14.42)	56(23.43)	
6–11	254(74.05)	79(75.96)	175(73.22)	
12–16	18(5.25)	10(9.62)	8(3.35)	
GA (weeks)				0.659
< 37	19(5.54)	4(3.85)	15(6.28)	
37–42	318(92.71)	98(94.23)	220(92.05)	
> 42	6(1.75)	2(1.92)	4(1.67)	
BW (g)				0.917
< 2500	8(2.33)	2(1.92)	6(2.51)	
2500–4000	317(92.42)	97(93.27)	220(92.05)	
> 4000	18(5.25)	5(4.81)	13(5.44)	
Mode of delivery				0.858
Vaginal	118(34.40)	37(35.58)	81(33.89)	
Cesarean	225(65.60)	67(64.42)	158(66.11)	
Abnormal birth history				0.009
No	243(70.85)	63(60.58)	180(75.31)	
Yes	100(29.15)	41(39.42)	59(24.69)	
With underlying diseases				0.201
No	138(40.23)	36(34.62)	102(42.68)	
Yes	205(59.77)	68(65.38)	137(57.32)	
**Parental factors, n (%)**				
Maternal age at pregnancy (years)				0.221
< 30	246(71.72)	79(75.96)	167(69.87)	
30–35	82(23.91)	19(18.27)	63(26.36)	
> 35	15(4.37)	6(5.77)	9(3.77)	
Method of feeding (within 6 months after birth)				0.114
Exclusive breastfeeding	167 (48.69)	47(45.19)	120(50.21)	
Formula feeding	84(24.49)	33(31.73)	51(21.34)	
Mixed feeding	92(26.82)	24(23.08)	68(28.45)	
Paternal education level				0.051
Primary	75(21.87)	31(29.81)	44(18.41)	
Secondary	69(20.12)	21(20.19)	48(20.08)	
University	199(58.02)	52(50.00)	147(61.51)	
Maternal education level				0.295
Primary	84(24.49)	31(29.81)	53(22.18)	
Secondary	56(16.33)	17(16.35)	39(16.32)	
University	203(59.18)	56(53.85)	147(61.51)	
**Family-environmental factors, n (%)**				
Family structure				0.265
Nuclear	145(42.27)	39(37.50)	106(44.35)	
Stem	180(52.48)	57(54.81)	123(51.46)	
Others(non-traditional)	18(5.25)	8(7.69)	10(4.18)	
Parenting pattern				0.000
Democratic	116(33.82)	21(20.19)	95(39.75)	
Slant scold	55(16.03)	24(23.08)	31(12.97)	
Slant interference	93(27.11)	26(25.00)	67(28.03)	
Indulgence	79(23.03)	33(31.73)	46(19.25)	
Parental involvement in care				0.114
Fully	170(49.56)	44(42.31)	126(52.72)	
Partially	106(30.90)	40(38.46)	66(27.62)	
None	67(19.53)	20(19.23)	47(19.67)	
Family relationship				0.003
Harmony	289(84.26)	78(75.00)	211(88.28)	
Inharmony	54(15.74)	26(25.00)	28(11.72)	
Family history				0.002
No TD/psychiatric disorders	317(92.42)	89(85.58)	228(95.40)	
Parent/close relatives with TD	15(4.37)	7(6.73)	8(3.35)	
Parent/close relatives with psychiatric disorders	11(3.21)	8(7.69)	3(1.26)	
**Clinical characteristics, n (%)**				
TD type				0.000
PTD	264(76.97)	62(59.62)	202(84.52)	
CTD/TS	79(23.03)	42(40.38)	37(15.48)	
Tic form				0.003
Simple motor/vocal tics	192(55.98)	45(43.27)	147(61.51)	
Complex tics	151(44.02)	59(56.73)	92(38.49)	
Tic severity				0.000
Mild	235(68.51)	53(50.96)	182(76.15)	
Moderate/Severe	108(31.49)	51(49.04)	57(23.85)	

### Unadjusted and adjusted logistic regression analysis

The unadjusted and adjusted logistic regression estimates of the associations between sociodemographic and clinical characteristics and behavioral problems are presented in Table 2. None of the variables was excluded by multiple collinearity diagnostics. The risk of behavioral problems in children with an abnormal birth history was higher than that in children with a normal birth history. Children with CTD/TS and moderate/severe tic severity also have an increased risk of behavioral problems. When more hypothetical confounding factors were controlled during the adjusted logistic regression analysis (Model 1 ~ 4), the effect of abnormal birth history (fully adjusted OR = 1.90, 95%CI 1.03 ~ 3.50, *P* = 0.040), CTD/TS (fully adjusted OR = 3.45, 95%CI 1.82 ~ 6.53, *P* < 0.001) and moderate/severe tic severity (fully adjusted OR = 2.13, 95%CI 1.18 ~ 3.84, *P* = 0.013) on the risk of behavioral problems remained consistent. Children aged 12–16 years (fully adjusted OR = 4.98, 95%CI 1.27 ~ 19.53, *P* = 0.021), with the parenting pattern of indulgence (fully adjusted OR = 2.17, 95%CI 1.00 ~ 4.69, *P* = 0.049) or parent/close relatives with psychiatric disorders (fully adjusted OR = 6.34, 95%CI 1.33 ~ 30.30, *P* = 0.021) increased the risk of behavioral problems, although slight variations in the point estimate and statistical significance were noted. The six variables were derived as follows: age, abnormal birth history, parenting pattern, family history, TD type and tic severity, and were included in the prediction model.

**Table 2:** Unadjusted and adjusted logistic regression analysis linking sociodemographic and clinical characteristics with behavioral problems in children with TD

	Unadjusted OR (95% CI)	Adjusted OR (95% CI)			
		Model 1	Model 2	Model 3	Model 4
**Personal factors**					
Female	0.64(0.35,1.18)	0.71 (0.38,1.34)	0.71(0.37,1.38)	0.77(0.39,1.52)	0.99(0.48,2.01)
Age (years)					
6–11	1.69(0.90,3.16)	1.72(0.90,3.28)	1.63(0.84,3.15)	1.60(0.80,3.21)	1.54(0.74,3.21)
12–16	4.67(1.57,13.89)	5.02(1.62,15.57)	4.27(1.32,13.85)	4.64(1.30,16.50)	4.98(1.27,19.53)
GA (weeks)					
37–42	0.60(0.19,1.85)	0.42(0.12,1.45)	0.41(0.11,1.44)	0.49(0.13,1.93)	0.42(0.10,1.82)
> 42	1.12(0.20,6.23)	1.50(0.26,8.54)	1.29(0.22,7.67)	1.78(0.30,10.70)	1.86(0.26,13.11)
BW (g)					
2500–4000	1.32(0.26,6.67)	1.06(0.19,6.02)	1.24(0.21,7.26)	1.08(0.17,6.75)	1.34(0.20,9.12)
> 4000	1.15(0.17,7.74)	0.88(0.12,6.68)	0.87(0.11,6.91)	0.66(0.07,6.01)	0.78(0.08,7.85)
Cesarean	0.93(0.57,1.50)	0.99(0.59,1.64)	0.96(0.57,1.62)	1.04(0.59,1.81)	1.08(0.60,1.94)
Abnormal birth history	1.99(1.22,3.24)	2.05(1.21,3.45)	2.15(1.26,3.67)	1.95(1.10,3.47)	1.90(1.03,3.50)
With underlying diseases	1.41(0.87,2.27)	1.29(0.78,2.13)	1.27(0.75,2.13)	1.15(0.66,1.99)	1.08(0.61,1.91)
**Parental factors**					
Maternal age at pregnancy (years)					
30–35	0.64(0.36,1.14)		0.62(0.34,1.14)	0.59(0.30,1.18)	0.51(0.25,1.07)
> 35	1.41(0.49,4.10)		1.48(0.47,4.68)	1.46(0.42,5.14)	1.60(0.41,6.17)
Method of feeding (within 6 months after birth)					
Formula feeding	1.65(0.95,2.87)		1.58(0.86,2.9)	1.75(0.91,3.38)	1.49(0.74,2.99)
Mixed feeding	0.90(0.51,1.60)		0.93(0.50,1.72)	1.04(0.54,1.99)	0.84(0.42,1.68)
Paternal education level					
Secondary	0.62(0.31,1.24)		0.56(0.24,1.32)	0.60(0.24,1.47)	0.64(0.24,1.70)
University	0.50(0.29,0.88)		0.43(0.18,1.06)	0.58(0.22,1.49)	0.59(0.21,1.63)
Maternal education level					
Secondary	0.75(0.36,1.53)		1.04(0.43,2.49)	1.04(0.41,2.61)	1.06(0.38,2.95)
University	0.65(0.38,1.12)		1.38(0.57,3.34)	1.09(0.43,2.77)	1.24(0.46,3.35)
**Family-environmental factors**					
Family structure					
Stem	1.26(0.78,2.04)			0.97(0.50,1.92)	0.75(0.36,1.56)
Others(non-traditional)	2.17(0.80,5.91)			0.96(0.29,3.14)	0.74(0.21,2.64)
Parenting pattern					
Slant scold	3.50(1.72,7.14)			2.02(0.84,4.87)	2.04(0.81,5.11)
Slant interference	1.76(0.91,3.38)			1.20(0.58,2.48)	1.21(0.56,2.60)
Indulgence	3.25(1.69,6.22)			2.1(1.01,4.33)	2.17(1.00,4.69)
Parental involvement in care					
Partially	1.74(1.03,2.92)			2.11(1.04,4.29)	2.11(1.00,4.48)
None	1.22(0.65,2.28)			1.18(0.50,2.76)	1.08(0.44,2.64)
Inharmony family relationship	2.51(1.39,4.55)			1.29(0.60,2.74)	1.19(0.53,2.67)
Family history					
Parent/close relatives with TD	2.24(0.79,6.36)			1.24(0.37,4.17)	1.14(0.31,4.18)
Parent/close relatives with psychiatric disorders	6.83(1.77,26.33)			7.09(1.59,31.56)	6.34(1.33,30.30)
**Clinical characteristics**					
CTD/TS	3.70(2.19,6.26)				3.45(1.82,6.53)
Complex tics	2.10(1.31,3.34)				1.21(0.67,2.19)
Moderate/Severe tic severity	3.07(1.89,5.00)				2.13(1.18,3.84)

Model 1 included personal factors only; Model 2 included personal and parental factors; Model 3 included personal, parental and family-environmental factors; Model 4 included personal, parental, family-environmental factors and clinical characteristics. All factors and associated estimates in Model 1 to Model 4 were listed

The first category of each categorical variable was chosen as the reference

### Development, validation and assessment of the prediction model

**Fig. 4:**
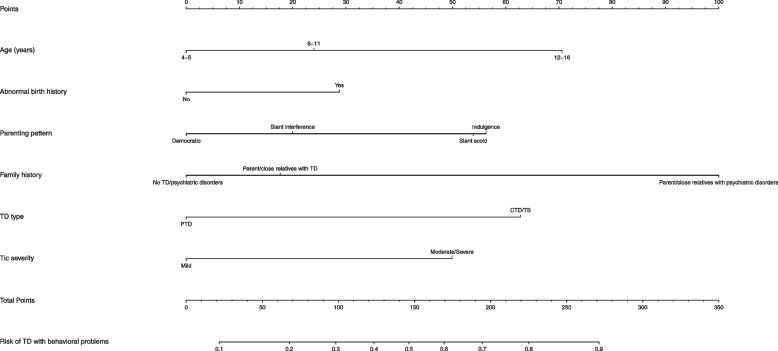
Nomogram for prediction of behavioral problems in children with TD. Each variable was projected upward to the value of the small ruler (points), to obtain the score for each parameter. The total score was calculated by adding each score from the small ruler. The higher the total score, the greater the likelihood of behavioral problems

**Fig. 5:**
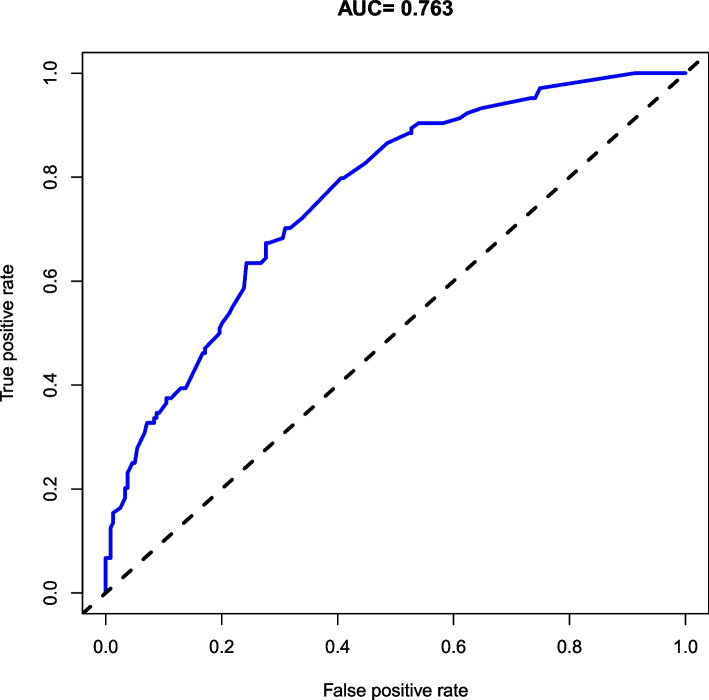
ROC curve of the nomogram for prediction of behavioral problems in children with TD

**Fig. 6:**
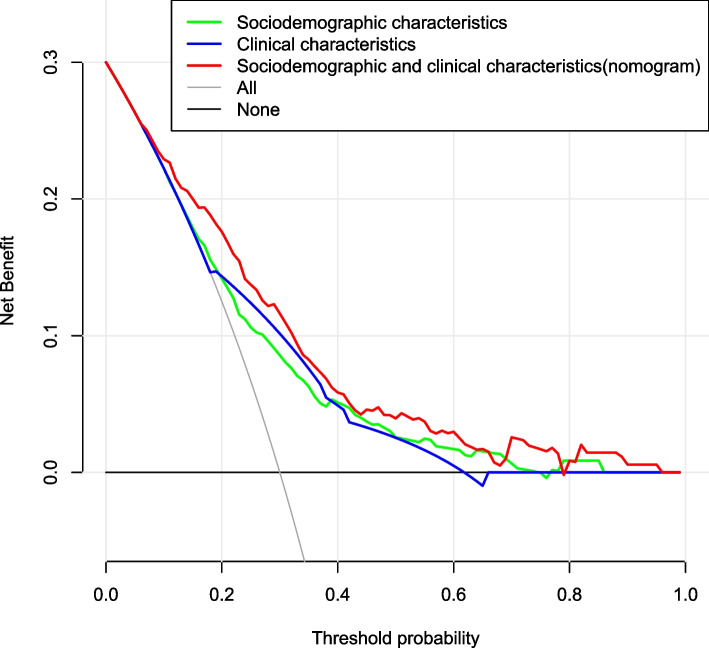
The DCA of the nomogram for prediction of behavioral problems in children with TD. The DCA showed that as the threshold probability was within the range from 0.08 to 1.0, this nomogram could adds more benefit than the scheme (either “treat none” or “treat all”). The nomogram also received a higher net benefit than either sociodemographic characteristics or clinical characteristics alone across the reasonable threshold probabilities

**Fig. 7:**
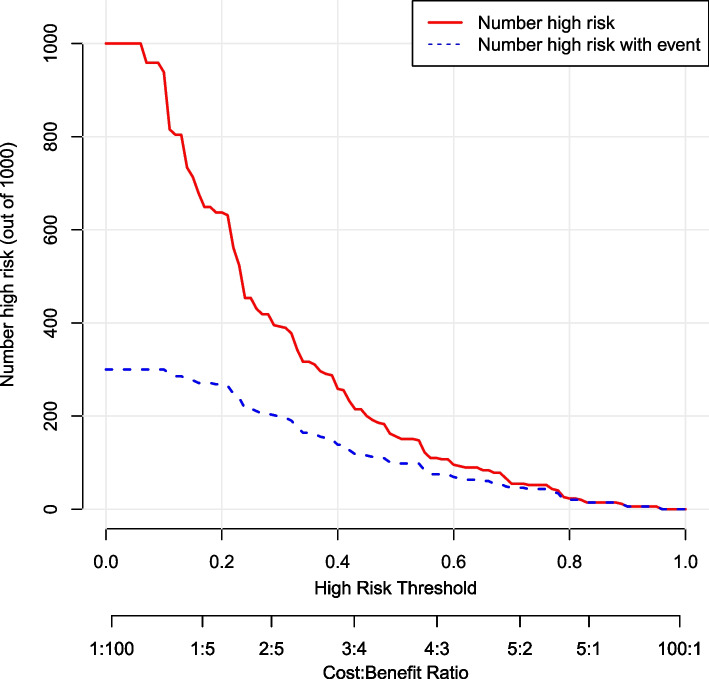
The CIC of the nomogram for prediction of behavioral problems in children with TD. The red curve indicates the number of people who are classified as positive (high-risk) by the prediction model at each threshold probability; the blue curve is the number of true positives at each threshold probability

The prediction model was constructed based on the six variables and is shown as a nomogram (Fig. 4). Each variable was projected upward to the value of the small ruler (points), to obtain the score for each parameter. The individualized risk of TD with behavioral problems can be calculated by summarizing the score of each variable point through the nomogram. The higher the total score, the greater the likelihood of TD with behavioral problems. Using bootstrapping, internal validation showed that the C-index of the nomogram was 0.763 (95%CI, 0.710 ~ 0.816). The ROC curve is shown in Fig. 5 (AUC = 0.763, equivalent to the C-index). The clinical utility of this nomogram was demonstrated by the DCA and CIC. DCA showed that as the threshold probability was within the range from 0.08 to 1.0, this nomogram could add more benefit than the scheme (either “treat none” or “treat all”). The nomogram also received a higher net benefit than either sociodemographic characteristics or clinical characteristics alone across the reasonable threshold probabilities (Fig. 6). CIC showed that the number of positive cases predicted by the nomogram was highly matched with the number of true-positive cases when the threshold probability was above 70% (Fig. 7).

## Discussion

Here, we describe the sociodemographic and clinical characteristics of children with TD and behavioral problems in a hospital-based population of children aged 4–16 years. Real-world studies are required to achieve a comprehensive understanding of the trajectories and management of this long-term condition. Herein, we developed a model to predict the individualized risk of behavioral problems among children with TD based on sociodemographic and clinical characteristics, and applied a nomogram to exhibit the combined impact of variables in the prediction model. The identified predictors were as follows: age 12–16 years, abnormal birth history, parenting pattern of indulgence, parent/close relatives with psychiatric disorders, CTD/TS and moderate/severe tic severity. The model showed good calibration and discrimination and was eligible for clinical practice according to the satisfactory results of the C-index, AUC of the ROC curve, DCA and CIC.

Behavioral problems were identified in 30.32% of the TD cases using the CBCL scale in this study. This rate was much higher than the prevalence of behavioral problems among primary and middle school students in Hubei province [27]. The relationships between individual factors, family factors, clinical characteristics and behavioral problems in children with different medical conditions were inconsistent [28–30]. However, sex effect was more exactly in surveys [31, 32]. This study sample contained a much higher proportion of males than females (male-to-female ratio close to 4:1 in our study), which is consistent with previous studies [1]. The gender differences have also been well documented in neurodevelopmental disorders in previous studies [33, 34]. In this study, male patients had a higher rate of behavioral problems than female patients, and the rate gradually significantly increased with age in the male patients, whereas the opposite trend was observed in the female patients. This may be related to sexual dimorphism in the maturation of neural networks. Previous studies have also reported that elevations in emotional reactivity and reward processing follow an inverted U-shape in terms of onset and remission, with a peak occurring during adolescence. Furthermore, sex-dimorphic activation patterns of enhanced left fronto-striatal activation in females and enhanced right parietal activation in males during motor inhibition appear to be the result of underlying gender differences in the functional maturation of these brain regions [35, 36].

Looking at personal factors, first, we found that children aged 12–16 years were more likely to have behavioral problems, and the overall age differences in the adjusted logistic regression analysis were nearly significant (*P* = 0.070). Although studies have shown that frequency and tic severity decline with age, social, peer and family relationships, abilities, and school/work impairment caused by tic decrease at follow-up do not completely improve [37]. Second, our study reported that TD cases with an abnormal birth history had a risk effect on behavioral problems when compared to TD patients with a normal birth history. The association between prenatal and perinatal epigenetic factors and TD has been reported in previous studies [9], indicating that prenatal and perinatal factors of abnormal birth history should be considered in the clinical spectrum of TD and comorbidities, which may share a common etio-pathogenetic basis.

Considering parental factors, it seemed that higher paternal education level was a protective factor against behavioral problems among TD cases, although the overall association between parental education level and behavioral problems was not significant in our study. This outcome is inconsistent with previous findings. Cui et al. previously investigated the risk factors of comorbid ADHD in children with TS, reporting that low family education and lower cultural levels of parents were key risk factors for the co-occurrence of TS and ADHD [38]. Hosokawa et al. found that lower maternal education level predicted externalized problems and behavioral problems, while paternal education level did not predict any clinically significant behavioral problems [39]. The lack of significance of the association in our study may be due to different sample populations, or affected by other factors which may act as confounders in the described relation between parental education level and behavioral problems. Because literature on this relation in children with TD is rare, further research mapping out the associations is needed.

Regarding family-environmental factors, as expected, the current study showed a positive association between the parenting pattern of indulgence and behavioral problems in children with TD. This result is consistent with those of previous studies [40, 41]. The epidemiological findings of children’s behavioral problems also indicated that lack of supervision, limited conversation time and parent–child interactions may contribute to problematic behaviors [42, 43]. Negative parenting styles foster a hostile and neglectful environment for children and inhibit their ability to appropriately self-regulate behavioral problems [43]. Therefore, it would be helpful to minimize the occurrence and development of behavioral problems in children with TD via providing proper suggestions for parents on parenting patterns in clinical practice. In addition, the findings in our study revealed the role of family history in TD and behavioral problems. TD are considered to be one category among the most heritable neuropsychiatric conditions. Comorbid symptoms such as ADHD, OCD, and depression persist into adulthood and require close monitoring for its heritability [9]. These findings should encourage clinicians and child-health practitioners to pay more attention to high-risk individuals.

The relationship between behavioral problems and clinical characteristics in children with TD was significant, which is consistent with our expectations. First, having moderate/severe tic severity increases the risk of behavioral problems. Children with CTD/TS also have an increased risk of behavioral problems. Previous studies have also reported the effect of clinical characteristics on TD with behavioral problems [11, 44]. This independent association could be explained in several possible ways: First, the more severe, complex or persistent the tics, the more obvious the symptoms and the greater the functional impairment will present [45]. Second, these children showed more problems in their peer relationships and were perceived as withdrawn and unpopular by their peers [10]. Third, such children nearly always present with multiple psychiatric comorbidities, which are often more impairing than the tics themselves [7, 46]. Complex tics were statistically significant in the unadjusted logistic analysis, but lost this association in the adjusted logistic analysis. Further studies are required to determine whether these findings can be replicated using larger datasets.

Moreover, this study developed a prediction model for the individualized risk of behavioral problems in children with TD, and a nomogram was plotted for the prediction model. To date, the application of a nomogram to predict the risk of TD with behavioral problems is lacking, although nomograms have been widely used as a reliable clinical tool to create a simple intuitive graph to quantify the risk of a clinical event of interest in other diseases [19, 20]. In the present study, the model based on age, abnormal birth history, parenting pattern, family history, TD type and tic severity had a significant predictive performance for behavioral problems in children with TD. This nomogram was feasible for making beneficial decisions in clinical practice, according to the satisfactory results of the DCA and CIC. This showed that a family history of parent/close relatives with psychiatric disorders accounted for the largest contribution to the risk of TD with behavioral problems, which further emphasizes the heritability of TD development. The contributions of the factors including age 12–16 years, CTD/TS, parenting pattern of indulgence, moderate/severe tic severity and abnormal birth history, though less than that of family history, indicate that clinical characteristics and other sociodemographic characteristics also contribute to the occurrence of TD with behavioral problems. This nomogram would allow clinicians to rapidly identify patients with a higher risk of behavioral problems and tailor necessary interventions as early as possible to improve clinical outcomes.

Although this study has some advantages, it also has several limitations. First, this was a hospital-based cross-sectional study, and all participants were recruited from one hospital, potentially leading to admission bias and therefore a relatively lower quality of evidence. Second, behavioral problems are complex and involve multiple factors. Residual confounding factors may have affected the associations, although we assessed numerous factors. Third, the outcome variable was integrated, and non-specific psychometrics was used in this study. Therefore, the clinical utility of distinguishing between specific psychopathologies is limited. Finally, the prediction model was internally validated in our study, and external validation is lacking. Future research involving large-scale, multicenter settings is required to further validate our findings of the study.

## Conclusions

In our study, nearly one-third of children with TD had behavioral problems. Age 12–16 years, abnormal birth history, parenting pattern of indulgence, parent/close relatives with psychiatric disorders, CTD/TS and moderate/severe tic severity, were probable predictors of behavioral problems in children with TD based on multivariate logistic regression analyses and these factors were used to develop a prediction model. The model shown as a nomogram had good discriminative ability and predictive performance for beneficial clinical decisions, according to the satisfactory results of the C-index, AUC, DCA and CIC. This model provides a comprehensive understanding of factors associated with behavioral problems in TD by visual graphs and allows clinicians to rapidly identify patients with a higher risk of behavioral problems and tailor necessary interventions to improve clinical outcomes.

## Data Availability

The datasets used and/or analyzed during the current study are available from the first or corresponding author on reasonable request.

## Abbreviations

ADHD: Attention-deficit/hyperactivity disorder
AUC: Area under curve
BW: Birth weight
CBCL: Child Behavior Checklist
CI: Confidence interval
CIC: Clinical impact curve
C-index: Concordance index
CTD: Chronic motor or vocal tic disorder
DCA: Decision curve analysis
DSM-5: Diagnostic and Statistical Manual of Mental Disorders, fifth Version
GA: Gestational age
OCD: Obsessive–compulsive disorder
ORs: Odds ratios
PTD: Provisional tic disorder
ROC: Receiver operating characteristic
TD: Tic disorders
TS: Tourette syndrome
YGTSS: Yale Global Tic Severity Scale
